# Anodic and Cathodic Extracellular Electron Transfer by the Filamentous Bacterium *Ardenticatena maritima* 110S

**DOI:** 10.3389/fmicb.2018.00068

**Published:** 2018-02-06

**Authors:** Satoshi Kawaichi, Tetsuya Yamada, Akio Umezawa, Shawn E. McGlynn, Takehiro Suzuki, Naoshi Dohmae, Takashi Yoshida, Yoshihiko Sako, Nobuhiro Matsushita, Kazuhito Hashimoto, Ryuhei Nakamura

**Affiliations:** ^1^Biofunctional Catalyst Research Team, Center for Sustainable Resource Science, RIKEN, Saitama, Japan; ^2^Department of Electronic Chemistry, Interdisciplinary Graduate School of Science and Engineering, Tokyo Institute of Technology, Tokyo, Japan; ^3^Earth-Life Science Institute, Tokyo Institute of Technology, Tokyo, Japan; ^4^Biomolecular Characterization Unit, Center for Sustainable Resource Science, RIKEN, Wako, Japan; ^5^Laboratory of Marine Microbiology, Graduate School of Agriculture, Kyoto University, Kyoto, Japan; ^6^Department of Materials Science and Engineering, Tokyo Institute of Technology, Tokyo, Japan; ^7^National Institute for Materials Science, Tsukuba, Japan

**Keywords:** extracellular electron transfer, filamentous bacteria, iron reducing bacteria, nitrate, cytochromes

## Abstract

*Ardenticatena maritima* strain 110S is a filamentous bacterium isolated from an iron-rich coastal hydrothermal field, and it is a unique isolate capable of dissimilatory iron or nitrate reduction among the members of the bacterial phylum *Chloroflexi*. Here, we report the ability of *A. maritima* strain 110S to utilize electrodes as a sole electron acceptor and donor when coupled with the oxidation of organic compounds and nitrate reduction, respectively. In addition, multicellular filaments with hundreds of cells arranged end-to-end increased the extracellular electron transfer (EET) ability to electrodes by organizing filaments into bundled structures, with the aid of microbially reduced iron oxide minerals on the cell surface of strain 110S. Based on these findings, together with the attempt to detect surface-localized cytochromes in the genome sequence and the demonstration of redox-dependent staining and immunostaining of the cell surface, we propose a model of bidirectional electron transport by *A. maritima* strain 110S, in which surface-localized multiheme cytochromes and surface-associated iron minerals serve as a conduit of bidirectional EET in multicellular filaments.

## Introduction

Redox gradients are generated by a variety of mechanisms in natural environments, and the electrical potential from these gradients can generate electric current, termed geoelectric current, when two such gradients are spatially segregated and electrically connected by a conduit. In some cases, these redox gradients are separated by large distances and can be dissipated by conductive ore bodies in the form of geoelectric currents (Sato and Mooney, 1960). Recently at a smaller scale than ore bodies, *in situ* experiments have demonstrated that chimney deposits of structured metal sulfide located at deep-sea hydrothermal field are capable of electrically bridging large redox potential which exists between the hot and reduced sulfide bearing hydrothermal fluid flowing within the chimney and the cool and oxic ambient sea water on the outside (Lowy et al., 2006; Nakamura et al., 2009b, 2010; Yamamoto et al., 2013, 2017; Ang et al., 2015; Rowe et al., 2015; Gartman et al., 2017).

In addition to the abiotically mediated currents mentioned above, geoelectric current derived from biotic metabolism in marine sediments has also been proposed (Nakamura et al., 2009b; Nielsen et al., 2010; Kondo et al., 2015.). For example, sediment dwelling uncultured multicellular filamentous bacteria of the family *Desulfobulbaceae* have been suggested to couple sulfide oxidation with oxygen or nitrate reduction over microbially large (>1 cm) distances (Nielsen et al., 2010; Pfeffer et al., 2012; Risgaard-Petersen et al., 2012; Schauer et al., 2014; Marzocchi et al., 2014; Larsen et al., 2015; Seitaj et al., 2015; Vasquez-Cardenas et al., 2015; Risgaard-Petersen et al., 2015; Sulu-Gambari et al., 2016; Burdorf et al., 2017). Such long-range redox coupling reactions seem to require metabolic synchronization and perhaps metabolic specialization amongst cells within the same filament, since cells on each terminus of the filament could experience very different environments from each other. However, the long-range electron transfer of the multicellular filamentous bacteria remains an open question due to their unculturability and unique structures of insulating surface (Pfeffer et al., 2012). Further, the hypothesis has been challenged by the direct measurement of centimeter-long electron transport through reduced marine sediments: they demonstrated electrical conductivities sufficient for the estimated electron fluxes without any filamentous microbes (Malvankar et al., 2015a,b).

**FIGURE 1:**
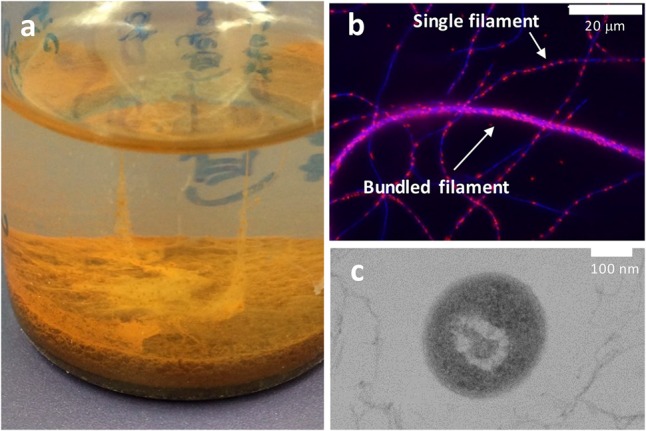
**(a)** Photograph showing a filamentous structure formed by strain 110S between the surface of the liquid medium and ferrihydrite precipitate. The liquid medium is approximately 2 cm in depth. **(b)** Single filaments and a bundled structure of strain 110S were visualized using an overlay of 4′,6-diamidino-2-phenylindole (DAPI, blue) staining and 5-cyano-2,3-di-p-toluyl-tetrazolium chloride (CTC, red) staining. Blue and red spots indicate the localization of double-stranded DNA and NAD(P)H, respectively. **(c)** Cross-sectional TEM image of strain 110S showing its smooth surface structure. Scale bars indicate 20 μm **(b)** and 100 nm **(c)**.

*Ardenticatena maritima* strain 110S is a facultative anaerobic dissimilatory iron-reducing thermophile that forms multicellular filaments composed of hundreds of 0.5 × 2 μm cells arranged end-to-end (Kawaichi et al., 2013a). It inhabits the sediment of a coastal hydrothermal field that contains steep physicochemical gradients (Kawaichi et al., 2013b). There, the exposure of reduced hot fluids to oxic seawater may provide microenvironments with different redox potentials in a narrow area. In static cultures of strain 110S, bundled filamentous structures exceeding 2 cm in length were observed between the liquid surface and precipitated iron minerals (**Figures 1a,b**). Furthermore, in contrast to the structure of the typical Gram-negative microorganisms such as *Geobacter* and *Shewanella, A. maritima* is presumably monoderm (Kawaichi et al., 2015) similar to the Gram-positive iron-reducer *Thermincola potens* (Wrighton et al., 2011; Carlson et al., 2012) (**Figure 1c**). For *Geobacter* and *Shewanella* species, bacterial outer-membrane (OM) multiheme *c*-type cytochromes (*c*-cyts) and/or filamentous conductive appendages, so called nanowire, have been identified to mediate extracellular electron transfer (EET) to the surface of Fe(III) oxides either directly or indirectly via electron shuttles (Reguera et al., 2005; Gorby et al., 2006; Ntarlagiannis et al., 2007; El-Naggar et al., 2010; McLean et al., 2010; Okamoto et al., 2012, 2013; Wegener et al., 2015). However, the unique surface structure of strain 110S suggested that if strain 110S is capable of performing anodic and cathodic EET using an insoluble electron accepter and donor, respectively, the mechanism for these processes could be distinct from previously described *Geobacter* and *Shewanella species.* It is also anticipated that if strain 110S is also capable of performing long-range electron transfer, the bundled filamentous structure could couple spatially segregated redox potentials to generate electrical current. In an effort to evaluate the potential of the multicellular filamentous bacteria to electrically bridge two redox environments, here we cultivated strain 110S in a three-electrode electrochemical cell (EC cell) and investigated the bidirectional EET ability by measuring generation of anodic and cathodic current. Additionally, to advance our understanding of the distant electron transfer, we identified possible heme binding proteins which could facilitate electron transfer and investigated their location within the filaments of strain 110S.

## Materials and Methods

### Bacterial Strain and Culture Conditions

*Ardenticatena maritima* strain 110S^T^ (NBRC 107679) was routinely cultivated at 60°C under aerobic conditions in liquid medium of 100 ml filtrated autoclaved Marine Broth 2216 (Difco) in a 300-ml conical flask. The suspension at an optical density at 600 nm (OD_600_) was centrifuged (5,000 × *g* for 5 min) and the cells were then washed with Marine Broth which lacks both ferric citrate and organic complex for three times prior to use in electrochemical experiments.

### Anodic and Cathodic Current Measurements

A three-electrode EC cell (8 ml capacity) was assembled using a platinum wire and Ag/AgCl/saturated KCl as counter and reference electrodes, respectively. An optically transparent conducting glass substrate [fluorine-doped tin oxide (FTO)-coated glass electrode, resistance: 20 Ω/square, size: 30 mm × 30 mm; SPD Laboratory, Inc.] with a surface area of 1.8 cm^2^ was placed on the bottom surface of the reactor as the working electrode. A 16-chanel potentiostat (VMP3; BioLogic) was used as an automatic polarization system. For the anodic EET experiments, 5 ml of electrolyte (autoclaved Marine Broth 2216 lacking ferric citrate, followed by 0.22-μm syringe filtration) was added to an EC cell and was then purged with pure-nitrogen gas to displace oxygen for >30 min. In this electrolyte, peptone (5 g/L) and yeast extract (5 g/L) serves as an electron donor. For the cathodic EET experiments, 5 ml of an autoclaved sodium chloride solution [3% (w/v)] was added to an EC cell as electrolyte and purged with pure-nitrogen for >30 min. Here, sodium nitrate (final concentration, 5 mM) was used as an electron acceptor and no organic substrates were added in the EC cell. In all electrochemical experiments, the reactor temperature was maintained at 60°C and no agitation was made during the measurements. To evaluate the effects of insoluble iron minerals on the current generation of strain 110S, ferrihydrite (2.5, and 10 mM), or hematite (10 mM) was added to the electrolyte, and anodic current was measured.

### UV/Vis Spectroscopy

Microbial cells were collected by centrifugation (3,000 × *g* for 5 min), re-suspended in filtrated fresh Marine Broth 2216 to an OD_600_ of 24, and then added into a Pyrex cell with an optical path length of 1 mm. The Pyrex cell was then mounted in front of an integrating sphere to measure diffuse light transmission (Nakamura et al., 2009a).

### Identification of Membrane-Bound *c*-cyts

To isolate membrane-bound proteins, strain 110S cells were sonicated and separated into soluble and insoluble fractions by centrifugation (20000 × g for 30 min). Proteins in each fraction (crude extract, soluble fraction, and insoluble fraction) were separated by SDS-PAGE. In-gel heme-staining was performed based on the peroxidase activity of *c*-cyt proteins. Bands specific to the insoluble fraction (Supplementary Figure S5) were excised and digested with a trypsin. The digested peptides were separated by nano ESI spray column (75 μm [ID] × 100 mm [L], NTCC analytical column C18, 3 μm, Nikkyo Technos) with a linear gradient of 0–35% buffer B (100% acetonitrile and 0.1% formic acid) for 10 min at a flow rate of 300 nL/min (EASY-nLC 1000 Liquid Chromatograph, Thermo Fisher Scientific). Eluted peptides were analyzed on line by tandem mass spectrometry. The mass spectrometer (Q Exactive mass spectrometer, Thermo Fisher Scientific) was operated in the positive-ion mode, and the MS and MS/MS spectra were acquired using a data-dependent TOP10 method (Michalski et al., 2011). The resulting data were searched against a protein database for strain 110S using Mascot Server software (Matrix Science) with variable modifications parameters [Acetyl (Protein N-term), Gln- > pyro-Glu (N-term Q), Deamidated (NQ), Oxidation (M), Propionamide (C)]. Detected proteins lacking a heme-binding motif, and a signal sequence were omitted and listed in **Table 1**.

**Table 1:** Putative heme proteins detected in the heme-stained bands of membrane fraction by LC-MS/MS.

Band Size (LC-MS/MS)	Locus_tag	AA seq. length	Predicted MW (by ExPASy)	Number of heme binding motif (CXXCH)	Signal peptide (by SignalP 4.1 Gram-Positive)	Number of transmembrane helices (by HMMTOP)^a^	Top hit^b^	*E* value
16	ARMA_0652	287	30749.6	2	N	1 (8-27)	Putative cytochrome *c* oxidase submit II [*Caldiinea aerophila* DSM 14535 = NBRC 104270]	4E-45
	ARMA2402	158	17912.9	1	N	2 (23-47, 60-84)	Hypothetical protein [*Thermomicrobium roseum*]	5E-43
	ARMA_2518	127	14202.5	1	N	1 (6-22)	Cytochrome *c* oxidase polypeptide II [*Caldiinea aerophila* DSM 14535 = NBRC 104270]	1E-40
16, 30	ARMA1484	344	37991.4	1	Y	3 (6-23, 48-66, 91-108)	Cytochrome *c* oxidase submit II [*Sphaerobacter thermoplilus*]	3E-81
30	ARMA_0541	155	16414.1	1	Y	1 (6-22)	Cytochrome *c* family protein [*Caldilinea aerophila* DSM 14535 = NBRC 104270]	2E-40
74	ARMA0580	430	47928.6	7	Y	1 (8-30)	Hypothetical protein [*Desulfosporosinus acidiphilus*]	3E-118
	ARMA_2517	1036	114866.7	1	N	0	Molybdopterin oxidoreductase [*Defenisoma canini*]	0

*AA, amino acid; MW, molecular weight. ^*a*^Numbers in parentheses indicate the AA position. ^*b*^Source organisms are shown in parentheses.*

### Immunostaining

Two polyclonal antibodies against ARMA_0580 protein were prepared by Sigma–Aldrich Japan (Tokyo, Japan). Briefly, two antigenic sites (amino acid numbers 148–161 and 350–385) were identified *in silico* based on the amino acid sequence derived from the genome sequence of *A. maritima* strain 110S. Antibodies were then generated against synthesized peptides in rabbits immunized with synthesized oligopeptides for each antigenic site conjugated with keyhole limpet hemocyanin as a carrier protein. Intact cells of strain 110S have been suspended in PBS buffer. Antiserum solutions containing the target antibodies were added to samples at final concentration of 1% (v/v). After overnight incubation at RT, the serum solution was removed, and the cells were washed three times with PBS. Fluorescein isothiocyanate (FITC)-labeled anti-rabbit IgG antibody solution (diluted 1:320 in PBS) was added to each sample, which were then further incubated for overnight at RT. The antibody solution was removed by washing 3 times with PBS, and the stained cells were collected by filtration for microscopic observation. Confocal images were acquired with a Zeiss LSM 710 microscope (Carl Zeiss Inc., Germany) equipped with an argon laser.

### TEM Sample Preparation and Observation

Cells were transferred with a pipet onto a glass slide in an area of approximately 2 mm in diameter and then overlaid with molten agar (2% noble agar in 100 mM HEPES buffer solution (pH 8.0) containing 20 g/L NaCl). The agar plug was removed from the glass slide after solidifying, and was then sliced into small pieces. Agar blocks with enrobed cells were then fixed with 2% paraformaldehyde and 1.25% glutaraldehyde in 87mM HEPES at pH 8 and 12.5g/L NaCl for 75 min on ice. After fixation, the filaments were washed five times with 1 ml of 100 mM HEPES buffer solution (pH 8) containing 20 g/L NaCl, followed by two additions and removals of 50 mM Tris. A solution of 3,3′-diaminobenzidine tetrahydrochloride (DAB; Sigma–Aldrich, St. Louis, MO, United States) was prepared in 1 M HCl using sonication to dissolve the DAB powder at a concentration of 0.053 mg/ml. The DAB solution was then diluted into 50 mM Tris-HCl (pH 8) to achieve a final concentration of 0.0015 g DAB/ml buffer. The solution was briefly sonicated and immediately filtered through a 0.22-μm syringe filter. H_2_O_2_ (30% aqueous stock) was added to the DAB solution to achieve a final concentration of 0.02%. The H_2_O_2_/DAB solution was added to the samples of sliced agar containing the microbial filaments and incubated on a rocker for 2.5 h at RT. A DAB solution prepared without H_2_O_2_ was added to parallel samples as a negative control to enable visualization of Osmium binding in the absence of the DAB reaction product. The DAB solution was removed by 5 washes with 100 mM HEPES buffer (pH 7.8). An OsO_4_ solution (1% in 100 mM HEPES buffer) was added to each sample for staining. The samples were washed by pelleting and re-suspension 3 × 1 ml with 50 mM Tris pH 8.0, then 3 × 1 ml with 50 mM HEPES pH 7.8. Finally the agar blocks were embedded into LR White Resin by treatment with a graded ethanol series (15 min each of 25%, 50, 75, 100, 100, 100%), followed by treatment with 50% LR White Resin and 50% ethanol on a rocker for 30 min. The samples were then transferred to 100% LR White Resin for 1 h on a rocker followed by replacement of the resin with fresh 100% LR white resin and further incubation at 56°C for 2 days for polymerization.

TEM of 200-nm thin sections was performed on an LEICA EM UC7 (Leica Microsystems, Germany) with a Histo Diamond knife (EMS). The sections were mounted on copper grids that had been briefly flamed and rinsed in water to make hydrophilic, and were examined and imaged by a JEOL JEM-1400 at 80 kV.

## Results

### Anodic and Cathodic EET

**FIGURE 2:**
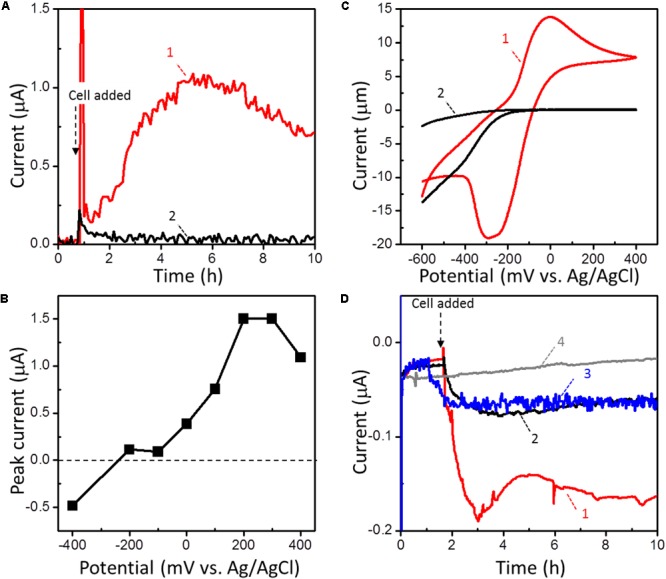
**(A)** Current (*I*) vs. time (*t*) measurements of microbial anodic current generation for intact (trace 1) and heat-killed (trace 2) strain 110S on an FTO electrode at +200 (mv vs. Ag/AgCl sat. KCl). Cell density was adjusted to OD_600_ = 1. **(B)** The potential dependence of the peak current for the microbial anodic current generation by strain 110S. **(C)** Whole-cell CVs (scan rate 1 mV/s) in the presence (trace 1) and absence (trace 2) of strain 110S cells. Cell density was adjusted to OD_600_ = 10. The cathodic current observed for the reactor without cells (trace 2) is not due to the reduction of oxygen, but likely attributable to the substrates present in Marine Broth 2216. **(D)** *I* vs. *t* measurements of microbial cathodic current generation by strain 110S on an FTO electrode at –100 mV. *I* vs. *t* curves was measured in the presence (trace 1) and absence (trace 2) of nitrate (5 mM) as an electron acceptor. Cell density was adjusted to OD_600_ = 2. Trace 3 is the current generation by heat-killed cells. The back ground current measured without any inoculation was also shown in trace 4.

We evaluated the anodic EET ability of strain 110S (OD_600_ = 1.0) cultivated anaerobically in a temperature-controlled electrochemical cell with a single chamber and three electrodes. Complex organic compound consisted of peptone and yeast extract and an FTO electrode was the sole electron donor and acceptor, respectively. At an applied potential of +200 mV (vs. Ag/AgCl sat. KCl), anodic current was generated immediately after cell inoculation at 60°C, and gradually increased over time (trace 1, **Figure 2A**). No current increase was observed following the addition of heat-killed cells, indicating that the observed catalytic current was generated by the metabolic activity of intact cells (trace 2, **Figure 2A**) (Repeat experiments on microbial current generation are available in Supplementary Figure S3). The anodic current displayed a clear potential dependency, and the onset and optimum potentials of the anodic EET of strain 110S were estimated to be in the range of -200 to -100 mV and +300 to +400 mV, respectively (**Figure 2B**). The observed onset potential was consistent well with the midpoint potential estimated from cyclic voltammetry of intact cells (CV; *E*_1/2_ = -150 mV; **Figure 2C**). The maximum current density (0.83 μA cm^-2^) obtained in the range of +300 to +400 mV is approximately 10-fold and 30-fold lower than that observed for *Shewanella oneidensis* and *Geobacter sulfurreducens cells*, respectively, cultured in the same electrochemical reactor used in the present experiments (Okamoto et al., 2013, 2014).

EET by strain 110S was also detected under cathodic conditions using an FTO electrode and nitrate as the sole electron donor and acceptor, respectively. The draft genome of strain 110S harbors a complete set of genes (*napAB, nirK, norBC*, and *nosZ*) for denitrification from nitrate to dinitrogen (Kawaichi et al., 2015). To maximize the cathodic EET, no electron donor (complex organic compound) was added in the EC cell. At an applied potential of -100 mV (vs. Ag/AgCl sat. KCl), the cathodic current was gradually increased after inoculation of the intact cells of strain 110S at 60°C to the EC cell (trace 1, **Figure 2D**). The generation of cathodic current was suppressed without nitrate (trace 2, **Figure 2D**), confirming that cathodic EET from the electrode to the bacterial filament was metabolically coupled with nitrate reduction. Heat-killed cells also generated a small cathodic current of approximately one third of the biotic current, and it gradually decreased over time but did not reach the baseline (trace 3, **Figure 2D**). This abiotic current may have been generated by heat-tolerant redox active molecules, such as free iron ions released from heme-containing proteins. The back ground current measured without any inoculation was approximately one fifth of the biotic current (trace 4, **Figure 2D**).

### Effects of Fe Minerals on EET

**FIGURE 3:**
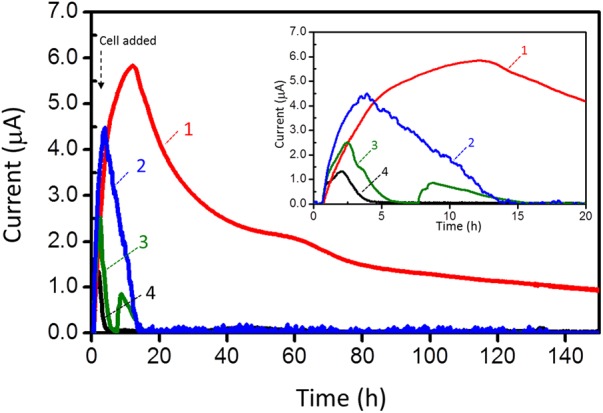
Anodic current (*I*) vs. time (*t*) measurements for 110S cultures containing ferrihydrite (trace 1: 10 mM, trace 2: 2.5 mM) and hematite (trace 3: 10 mM). Current generation in the absence of iron oxides was also shown in trace 4. Electrode potential was +200 mV. Cell density was adjusted to OD_600_ = 2.

Since strain 110S inhabits an iron-rich sediment and has the ability to reduce extracellular iron minerals (Kawaichi et al., 2013a), we evaluated its EET ability in the EC cell with hematite and ferrihydrite. In this experiment, ferrihydrite (2.5 and 10 mM) or hematite (10 mM) was added to the EC cell, and generation of anodic current was measured with a poised potential of +200 mV in the presence of complex organic compounds. The addition of hematite and ferrihydrite significantly increased anodic current generated by strain 110S (**Figure 3**). After reaching the current maxima, the current decreased gradually, which could be correlated with the decrease in the amount of organic substrates (electron donor), since a re-injection of complex organic compound resulted in the recovery of microbial current (Supplementary Figure S1). It was also confirmed that the enhanced anodic current generation is due to the synergetic effect of microbial respiration and iron oxides, as neither the addition of ferrihydrite nor hematite alone generates anodic current (Supplementary Figure S2). In addition to the enhanced current generation, the duration of current generation was extended with increasing amounts of added ferrihydrite. Compared to hematite, ferrihydrite had a greater effect on both the amount and length of current generation. In particular, strain 110S inoculated in the EC cell with 10 mM ferrihydrite was capable of maintaining current generating over 150 h (**Figure 3**, trace 1), which is distinct from the EC cells lacking ferrihydrite where current rapidly decreased and reached zero after 5 h of inoculation (**Figure 3**, trace 4) (Repeat experiments on microbial current generation in the presence of ferrihydrite are available in Supplementary Figure S3).

After inoculating strain 110S to the EC cell, the cells were stained with 5-cyano-2,3-di-p-toluyl-tetrazolium chloride (CTC) and 4′,6-diamidino-2-phenylindole (DAPI) to count viable cells. The proportion of respiring cells estimated by the number of CTC-stained cells divided by DAPI-stained cells detected in the presence of 10 mM ferrihydrite was 51.3 % and 33.6% after 20 and 140 h of inoculation, respectively. Those values were larger than those observed for the cells cultivated without ferrihydrite (17.8 and 0 % after 20 and 140 h, respectively). Thus, the enhanced anodic current suggests that the addition of ferrihydrite increased the number of viable cells in the multicellular filament which is capable of maintaining respiratory activity via electron transport to a distant electrode.

The onset potential for current generation was estimated from the potential-dependent current generation curves (-200 mV to -100 mV, **Figure 2B**) and CV (**Figure 2C**). It is consistent well with the redox potential of ferrihydrite which ranges between -100 and 100 mV vs. SHE (-300 to -100 mV vs. Ag/AgCl sat. KCl), whereas that of hematite is more negative (-287 mV vs. SHE [-487 mV vs. Ag/AgCl sat. KCl]) (Thamdrup, 2000; Straub et al., 2001). Thus, the enhanced anodic EET by ferrihydrite suggests that strain 110S cells utilize cell-surface associated ferrihydrite as a conduit of electron transport via a multistep redox hopping mechanism, as was observed for *Shewanella* species (Nakamura et al., 2009b, 2013). Although ferrihydrite is known to be a poor electron conductor, its ability for transporting electron is improved by strain 110S due to the microbial reduction of ferrihydrite (Supplementary Figure S4) [Direct electrochemical reduction of ferrihydrite or hematite was excluded, as no cathodic current was observed in the absence of bacterial cells (Supplementary Figure S2)]. It is therefore expected that the cell-surface associated ferrihydrite increased the number of cells in the multicellular filments that can participate in electrical current generation.

### Conductive Measurement of Intact Microbial Filaments

To measure the conductivity of the filamentous structures of strain 110S, whole cells were washed to remove the culture medium and extracellular molecules, and were then mounted on a two-electrode system consisting of micro-fabricated platinum electrode deposited on a fused quartz substrate (Supplementary Figure S4). To minimize damage to the microbial filaments, the samples were not chemically treated or subjected to critical-point drying. Cells were mildly dried under the continuous flow of 100% nitrogen at room temperature to maintain anaerobic conditions. However, our attempt to get reliable data for the conductivity of intact cells of strain 110S ended in failure due to the low electrical conductivity of the filaments.

### Multiheme Cytochromes

In the Gram-negative bacterium *Shewanella oneidensis*, multiheme *c*-cyts in bacterial outer-membrane have been identified to mediate EET to the surface of Fe(III) oxides and electrodes either directly or indirectly via electron shuttles (Myers and Myers, 1992; Magnuson et al., 2001). Multiheme *c*-cyts are also localized along the surface of conductive nanowires, which appear to be extensions of the outer membrane and periplasm (Pirbadian et al., 2014). The localization of *c*-cyts to these membrane extensions provides the most compelling evidence to date for long-range EET, and supports a proposed mechanism of multistep redox hopping (Pirbadian and El-Naggar, 2012) by which electrons are transported along a network of heme cofactors within microbial membrane filaments. Meanwhile, type IV pili and the multiheme *c*-cyts are identified to mediate long-range electron transfer for *Geobacter* species (Malvankar et al., 2011; Vargas et al., 2013). Here we speculated that multiheme *c*-cyts play a role in the electricity generation of strain 110S filaments, and attempted to detect cell-surface multiheme *c*-cyts in this strain using a multidisciplinary approach involving spectroscopy, genetics, biochemistry, and microscopy.

**FIGURE 4:**
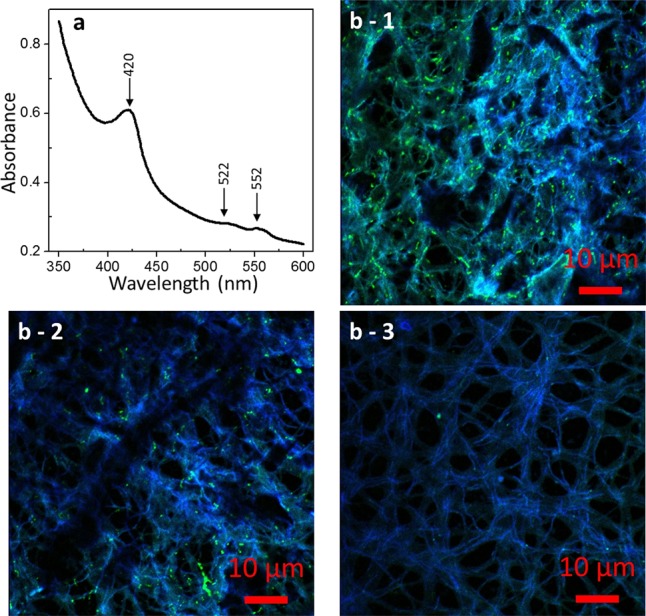
Microscopic images of strain 110S. **(a)** UV/Vis spectrum of a whole cell suspension of strain 110S. Absorbance peaks for the Soret band (420 nm) and Q-band (522 and 552 nm) of *c*-cyts were observed. **(b)** Confocal micrographs of strain 110S stained with pos_148-161 **(b-1)** and pos_350-365 **(b-2)** FITC-labeled polyclonal antibodies against ARMA_0580 protein, or without primary antibody **(b-3)**. Scale bars indicate 10 μm.

Because of the large molar absorption coefficient of heme irons, UV/Vis spectroscopy is a desirable method for investigating multiheme *c*-cyts in intact cells. To eliminate spectral interference from light scattered by the cell surface, diffuse transmission UV/Vis spectroscopy was performed. In the obtained diffuse transmission spectrum of strain 110S (**Figure 4a**), an intense absorption band at 420 nm (the Soret band) and weak absorption bands at 522 and 552 nm (the Q band) were observed. These peak positions are characteristic of the reduced form of heme groups of *c*-cyts (Higuchi et al., 1987; Okamoto et al., 2009). Considering the molar absorption coefficient of the Soret band is an order of 10^5^ (Solomon et al., 1992), the concentration of the heme groups in the cell suspension was estimated to be approximately 0.01 mM, a value that is 50 times lower than that of *Shewanella* cells (0.5 mM) (Nakamura et al., 2009a) with the same cell density.

### Multiheme Cytochrome Genes in the Genome

In the draft genome sequence of strain 110S (Kawaichi et al., 2015), 39 coding sequences (CDSs) with heme-binding peptide motifs (CXXCH) (Supplementary Table S1), including nine CDSs for multiheme proteins, were detected among the total of 3,355 predicted CDSs. Among the putative multiheme proteins, four sequences (ARMA_580, ARMA_1428, ARMA_1572, and ARMA_2257) contained a signal peptide sequence, which is necessary for translocation of the protein across the membrane and onto the cell surface. Notably, ARMA_580 contained the highest number (seven) of heme-binding motifs among the putative heme-proteins of strain 110S. A protein BLAST search revealed that this seven-heme protein is most similar to an unidentified hypothetical protein of the Gram-negative bacterium *Desulfosporosinus acidiphilus*, which is predicted to have iron oxidation/reduction activity based on functional genome distribution analysis (Altermann, 2012, 2014).

Cells of strain 110S are stain Gram negative and have a multilayer-like structure consisting of two electron-dense and two electron-transparent layers, as revealed by transmission electron microscopy (TEM) observation (Kawaichi et al., 2013a). However, they are thought to be monoderm, since biochemical and genomic analyses have shown that *Chloroflexi* lack lipopolysaccharide (LPS) and a number of corresponding biogenesis genes required for the formation of a double membrane (Sutcliffe, 2010, 2011). In the draft genome sequence of strain 110S, only a few CDSs involved in LPS biogenesis were detected, indicating that this pathway is also defective. In addition, the genome lacked CDSs for the BamA family of proteins, which plays a crucial role in outer membrane biogenesis (Knowles et al., 2009). These data support the monoderm feature of strain 110S. Since we could not isolate outer membrane proteins from cells of strain 110S using a preparation method developed for the model Gram-negative bacterium *Geobacter sulferreducens* (Inoue et al., 2010), sonicated crude cellular extracts were simply separated into soluble and insoluble fractions by centrifugation as an alternative method. Three protein bands with molecular masses of 16, 30, and 74 kDa were specifically detected in the insoluble membrane fraction by in-gel heme staining of SDS-PAGE based on the activity of *c*-cyt heme peroxidase (Supplementary Figure S5) (Goodhew et al., 1986). Using in-gel tryptic digestion and LC-MS/MS, the bands of 16- and 74-kDa heme-stained were identified as ARMA_0652 and ARMA_0580, which is consistent with the prediction above as a heme binding protein with signaling peptide sequence, respectively (**Table 1**). ARMA_0652 is a hypothetical protein with a sequence similar to the putative cytochrome *c* oxidase subunit II of *Caldilinea aerophila*.

To confirm the expression of multiheme *c*-cyts of strain 110S, we performed immunofluorescence microscopy using two polyclonal antibodies targeting ARMA_580, a surface-localized protein with putative seven heme binding. Consistent with the findings from the draft genome analyses, confocal microscopy of bacterial filaments with the primary and secondary antibodies showed FITC fluorescence derived from antibody binding (**Figures 4b-1,2**). Not all the cells in multicellular filaments were stained by the immunostaining, indicating a functional differentiation or a heterogeneous activity in the filament. This green fluorescence was not observed when the primary antibodies were omitted (**Figure 4b-3**), nor was a signal observed in control experiments using *E. coli* (Supplementary Figure S6).

**FIGURE 5:**
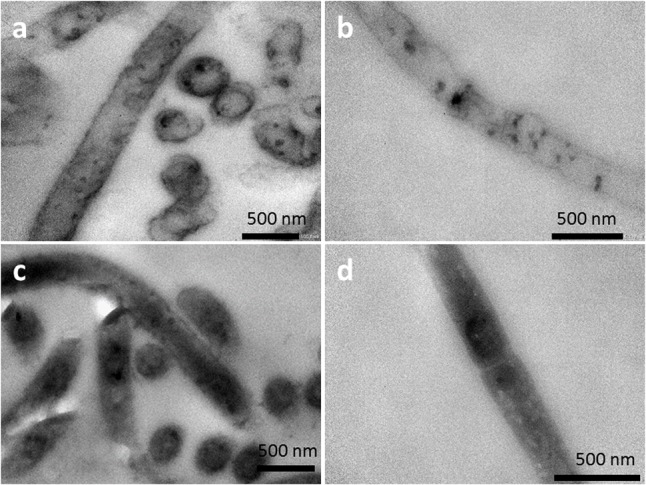
TEM images of DAB-stained cells **(a,b)** cultivated under anodic EET conditions. Image **(a,b)** was obtained in the presence and absence of H_2_O_2_, respectively. As a comparison to electrochemical cultivation, TEM images of DAB-stained cells cultivated anaerobically in a conical flask with LB were shown in **(c)** and **(d)**. Image **(c)** and **(d)** was obtained in the presence and absence of H_2_O_2,_ respectively.

The possibility of heme binding proteins on the cell surface was also investigated by a TEM technique suitable for detecting heme groups (Roels, 1974; Litwin, 1982). Aerobically grown cells of strain 110S were placed in an EC cell at an applied potential of +200 mV, fixed with glutaraldehyde, and treated with 3,3′-diaminobenzidine (DAB) followed by post staining with OsO_4_ (Seligman et al., 1968; McGlynn et al., 2015). TEM analysis of ultrathin sections revealed that a thin dark layer was present on the filament surfaces (**Figure 5a**), indicative of the presence of heme binding proteins at the cell surface of bacterial filaments (Roels, 1974; Litwin, 1982; McGlynn et al., 2015). This staining – derived from DAB oxidation catalyzed by heme with H_2_O_2_ – was not observed when DAB was included in the absence of H_2_O_2_ (**Figure 5b**). Thus, the contrast between TEM images with and without H_2_O_2_ suggests the location of heme within the filaments of strain 110S. As a comparison, DAB staining experiments were also conducted to the cells cultivated anaerobically in a conical flask with LB (**Figures 5c,d**). A thin layer of stained material was again observed on the surface when H_2_O_2_ was included. On the other hand, the treatment without H_2_O_2_ was much darker than the anodically (anaerobically) grown cells (compare **Figure 5b** and **Figure 5d**), likely due to the activity of a cytochrome c-oxidase protein which may catalyze DAB oxidation even in the absence of H_2_O_2_ (Seligman et al., 1968).

## Discussion

In the present study, we investigated the EET ability and the existence of surface-associated multiheme cytochromes of intact filaments of a pure-cultured filamentous bacterium. We provided evidence that *A. maritima* strain 110S has the ability to generate anodic and cathodic current when coupled with the oxidation of organic compounds and nitrate reduction, respectively (**Figure 2**). We also genomically identified seven putative heme binding *c*-cyts in strain 110S, one of them was shown to be expressed, and heme-binding protein(s) was suggested to be localized on the surface of strain 110S filaments with monoderm structure (**Figures 4, 5**). The metabolic rate of strain 110S calculated from the peak current of the andic EET at +200 mV and the cell density (10^2^ electrons per cell per second, which corresponds to approximately 10^4^ electrons per filament per second, **Figure 2A**) is four orders of magnitude lower than that of *S. oneidensis* MR-1 (10^6^ electrons per cell per second) (El-Naggar et al., 2010). However, this microorganism appears to have increased the EET ability by organizing filaments into bundled structures (**Figures 1a,b**), a possible strategy for achieving a *c*-cyt density allowing electron percolation along entire filaments (Broadbent and Hammersley, 1957). Additionally, the clustering of microbially reduced and conductive iron oxide minerals on the cell surface of strain 110S also facilitated electron transfer in the natural environment through self-constructed bacterial networks (Nakamura et al., 2009b, 2013), which may allow strain 110S to inhabit an iron-rich sediment with the ability to reduce extracellular iron minerals (Kawaichi et al., 2013b). Consistent with this speculation, ferrihydrite greatly increased both the maximum current generation and dulation of current generation by strain 110S (**Figure 3** and Supplementary Figure S3), which in turn resulted in the large increase in the number of viable cells in the multicellular filament. From those results we suggest that cell surface-associated iron-oxide minerals facilitated long-range electron transfer between the electrode and bacterial cells that were not directly in contact with the electrode.

**FIGURE 6:**
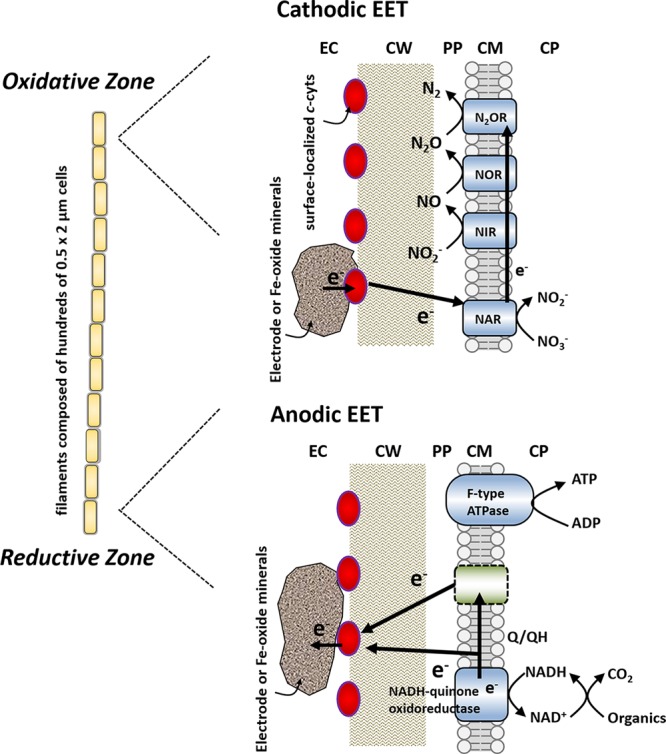
Proposed model for bidirectional extracellular electron transfer by strain 110S. Cells in the oxidative zone uptake electrons from the surface-localized conduit (*c*-cyts or conductive minerals) coupled with the half reaction of nitrate reduction. Simultaneously, cells localized in the reductive zone transfer electrons to the conduit coupled with the oxidative half reaction of organic compounds. Red ovals depict surface-localized *c*-cyts. EC, extracellular environment; CW, cell wall; PP, periplasm; CM, cellular membrane; CP, cytoplasm; NAR, nitrate reductase; NIR, nitrite reductase; NOR, nitric oxide reductase; N_2_OR, nitrous oxide reductase. Membrane-bound enzymes shown in blue (NAR, NIR, NOR, N_2_OR, NADH-quinone oxidoreductase, and F-type ATPase) are detected in the genome sequence. The location of nitrate reduction enzymes is modeled based on the situation with other monoderm (Gram positive) bacterium, *Bacillus azotoformans* (Suharti et al., 2004; Suharti and de Vries, 2005).

On the basis of the anodic and cathodic EET and the existence of surface-associated multiheme cytochromes, we propose a model of bidirectional EET by the filamentous bacterium *A. maritima* 110S, as schematically illustrated in **Figure 6**. In this model, cells localized in the reductive zone transfer electrons to the surface-localized conduit (*c*-cyts or conductive minerals) coupled with the oxidative half reaction of organic compounds (anodic EET). Simultaneously, cells in the oxidative zone uptake electrons from the extracellular insoluble electron donor coupled with the half reaction of nitrate reduction via the denitrification pathway (*napAB, nirK, norBC*, and *nosZ*) (cathodic EET). Thus, the ability of anodic and cathodic EET facilitate the interface of cellular metabolism with the segregated biogeochemical reactions in the sediments of a coastal hydrothermal field (Kawaichi et al., 2013b). Although the present study demonstrated the ability of the multicellular filamentous bacteria to conduct bidirectional EET for dissipating spatially segregated redox potentials in a form of electrical current, no direct evidence for the long-range electron transport along the filament was provided. Further work including the conductivity measurements along the filament and the establishment of a gene manipulation system for strain 110S will enable us to detect long-range electrical currents mediated by filamentous bacteria, which will lead to understand the impact of bio-geoelectric currents on chemical cycling.

## Author Contributions

RN, SK, TYa, and KH and conceived and designed the experiments. SK, TYa, AU, TS, ND, SM, and TYo performed the experiments. RN, SK, TYa, AU, TS, ND, SM, NM, TYo, and YS analyzed the data. RN, SK, TYa, and SM wrote the manuscript.

## Conflict of Interest Statement

The authors declare that the research was conducted in the absence of any commercial or financial relationships that could be construed as a potential conflict of interest.
