# Supplementary material for: Anodic and Cathodic Extracellular Electron Transfer by the Filamentous Bacterium Ardenticatena maritima 110S
Source: Front Microbiol. 2018 Feb 6;9:68. doi: 10.3389/fmicb.2018.00068 (PMC5808234; doi:10.3389/fmicb.2018.00068)
Supplement: Supplementary file 1 [file Data_Sheet_1.DOCX]

Supplementary Material

**Anodic and Cathodic Extracellular Electron Transfer by the Filamentous Bacterium *Ardenticatena maritima* 110S**

**Authors:** Satoshi Kawaichi^1 †⊥^, Tetsuya Yamada^1, 2⊥^, Akio Umezawa, Shawn McGlynn, Takehiro Suzuki Naoshi Dohmae, Takashi Yoshida, Yoshihiko Sako, Nobuhiro Matsushita, Kazuhito Hashimoto, and Ryuhei Nakamura *

*** Correspondence:** Corresponding Author: ryuhei.nakamura@riken.jp

^⊥^ These authors contributed equally.

# Supplementary Figures and Table

**Figure S1.** Current (*I*) vs. time (*t*) measurements of current generation by 110S on an FTO electrode in the presence (black line) and absence (grey line) of ferrihydrite. Marine broth (5 ml) was supplemented into the electrochemical reactor at the time points indicated with arrows. The electrode potential was +200 mV vs. Ag/AgCl.

**Figure S2.** Current (*I*) vs. time (*t*) measurements for the electrochemical reactor with 10 mM ferrihydrite and 10 mM hematite at +200 mV vs. Ag/AgCl. No bacterial cells were inoculated to the electrochemical cell. Neither ferrihydrite nor hematite alone generate current. Thus, the anodic EET observed in Fig. 3 in the main text was attributed to the respiratory activity of strain 110S.


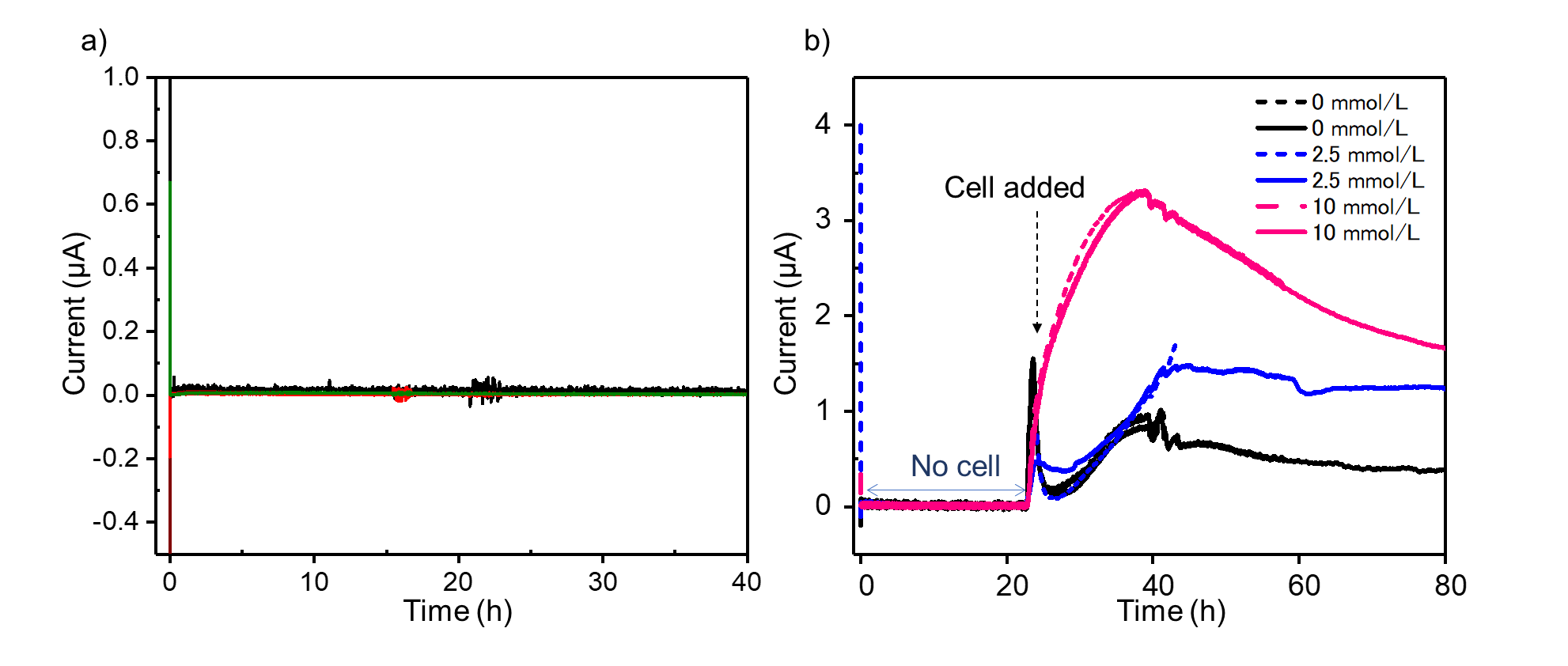


**Figure S3.** Repeat experiments on microbial current generation by strain 110S in the presence and absence of ferrihydrite. The electrode potential was +200 mV vs. Ag/AgCl. Two duplicated experiments (solid and broken line) showed essentially the same *I*- *t* curves.

**Figure S4** Current-voltage (I-V) curve measured by a two electrode system for conductive measurement. Red line: 110S cells cultivated with ferrihydrite. Black line: ferrihydrite without cells.

Experimental: The suspended samples of ferrihydrite with and without bacterial cells (20 μl) spread on a thin agarose gel (1% in ultra-pure-water). The agarose gel was then mounted face-down on a fused quartz substrate onto which platinum (Pt) terminals were deposited (ALS Co., Ltd, Japan) (The interelectrode distances were 210 µm). The strain 110S was grown in culture with ferrihydrite as electron acceptor and collected from 2ml culture. The cells were washed twice with ultra-pure water. The mounted cells were dried for 30 min in a desiccator under a continuous flow of pure N_2_ gas at room temperature (RT). Current-voltage (I-V) sweeps between -200 and +200 mV were performed at ambient conditions (The scan rate of 1 mV/s)


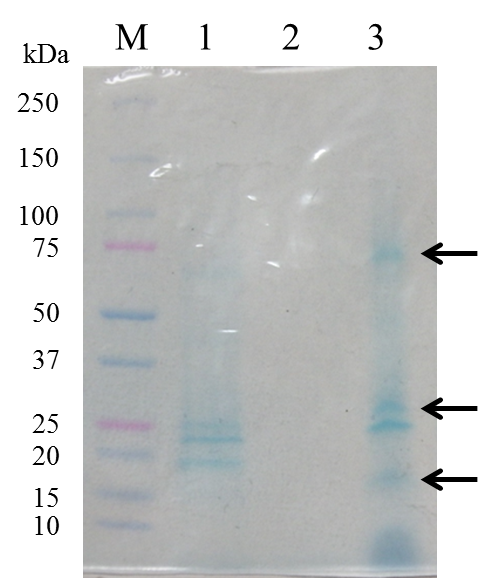


**Figure S5**. SDS-PAGE showing heme-proteins. Lane M, molecular weight marker (Precision Plus Protein Dual Color standards, Bio-Rad, USA); lane 1, crude extract; lane 2, soluble fraction; lane 3, insoluble fraction. Arrows indicate heme-protein bands extracted and further applied to LC-MS/MS analysis.

**Figure S6.** Confocal micrographs of strain 110S and *E. coli* stained with pos_148-161 and pos_350-365 FITC-labeled polyclonal antibodies against the ARMA_0580 protein, or without primary antibody. PBS containing 1% skim milk was used as a blocking reagent for immunostaining. Detector gain was lowered to diminish background fluorescence. Scale bars indicate 10 μm.
